# Genome-Wide DNA Methylation Changes of Perirenal Adipose Tissue in Rabbits Fed a High-Fat Diet

**DOI:** 10.3390/ani10122213

**Published:** 2020-11-26

**Authors:** Jiahao Shao, Xue Bai, Ting Pan, Yanhong Li, Xianbo Jia, Jie Wang, Songjia Lai

**Affiliations:** 1 College of Animal Science and Technology, Sichuan Agricultural University, Chengdu 611130, China; shaojh1997@163.com (J.S.); baixue333work@163.com (X.B.); lyh81236718@163.com (Y.L.); jaxb369@sicau.edu.cn (X.J.); wjie68@163.com (J.W.); 2 College of Veterinary Medicine, Sichuan Agricultural University, Chengdu 611130, China; panting555666@163.com

**Keywords:** DNA methylation, high-fat diet, rabbits

## Abstract

**Simple Summary:**

Obesity is spreading rapidly in most countries and regions, becoming a considerable public health concern because it is associated with type II diabetes mellitus, fatty liver disease, hypertension, and even certain cancers. The biological effects of caloric restriction are closely related to epigenetic mechanisms, including DNA methylation. Here, rabbits were used as a model to study the effect of a high-fat diet on the DNA methylation profile of perirenal adipose tissue. The results indicate that 2906 genes associated with differentially methylated regions were obtained and were involved in the PI3K-AKT signaling pathway (KO04151), linoleic acid metabolism (KO00591), DNA replication (KO03030), and MAPK signaling pathway (KO04010). In conclusion, high-fat diet may cause changes in the DNA methylation profile of adipose tissue and lead to obesity.

**Abstract:**

DNA methylation is an epigenetic mechanism that plays an important role in gene regulation without an altered DNA sequence. Previous studies have demonstrated that diet affects obesity by partially mediating DNA methylation. Our study investigated the genome-wide DNA methylation of perirenal adipose tissue in rabbits to identify the epigenetic changes of high-fat diet-mediated obesity. Two libraries were constructed pooling DNA of rabbits fed a standard normal diet (SND) and DNA of rabbits fed a high-fat diet (HFD). Differentially methylated regions (DMRs) were identified using the option of the sliding window method, and online software DAVID Bioinformatics Resources 6.7 was used to perform Gene Ontology (GO) terms and KEGG (Kyoto Encyclopedia of Genes and Genomes) pathway enrichment analysis of DMRs-associated genes. A total of 12,230 DMRs were obtained, of which 2305 (1207 up-regulated, 1098 down-regulated) and 601 (368 up-regulated, 233 down-regulated) of identified DMRs were observed in the gene body and promoter regions, respectively. GO analysis revealed that the DMRs-associated genes were involved in developmental process (GO:0032502), cell differentiation (GO:0030154), and lipid binding (GO:0008289), and KEGG pathway enrichment analysis revealed the DMRs-associated genes were enriched in linoleic acid metabolism (KO00591), DNA replication (KO03030), and MAPK signaling pathway (KO04010). Our study further elucidates the possible functions of DMRs-associated genes in rabbit adipogenesis, contributing to the understanding of HFD-mediated obesity.

## 1. Introduction

From the last 5 decades, the incidence of obesity has sharply increased, becoming one of the most considerable threats to human health because it is associated with the risk of type II diabetes mellitus, fatty liver disease, hypertension, and even certain cancers [1]. Obesity is a multifactorial pathological process, and genetic, environmental, and behavioral factors influence the development of obesity [2]. Nowadays, an imbalance between energy intake and expenditure is a major contributor to fat deposition in individuals predisposed to obesity [3]. Fat deposition is characterized by an increase in the number and size of adipocytes, and its process is closely related to physiological homeostasis, far beyond simple fat storage [4]. HFD has been shown to induce obesity in animal models and humans, and further induce a variety of obesity-related clinical diseases, such as osteoporosis, inflammation, and even neurodegeneration [5,6,7]. Perirenal fat, as part of abdominal visceral fat, is often used to elucidate the molecular and pathophysiological mechanisms of metabolic disorders associated with obesity or adipose development, because it is closely related to kidney injury, metabolism of triacylglycerol, and other metabolic regulation [8]. For example, detailed studies have shown that the perirenal fat thickness in obese patients could be a valuable marker to define the risk of developing hypertension and kidney dysfunction [9,10]. The expression profile of perirenal fat microRNA was changed during different growth stages of rabbits, and the differential microRNA expression was enriched for the MAPK signaling pathway, Wnt signaling pathway, aldosterone synthesis, and secretion pathways [11].

First proposed by Waddington in 1942, epigenetics refers to heritable changes in gene expression without an altered DNA sequence [12]. Epigenetics is caused by the interaction of environmental factors and intracellular genetic material, such as dietary factors, microRNA, and genomic imprinting, etc. Noteworthily, the biological effects of caloric intake are closely related to epigenetic mechanisms, including chromatin remodeling and DNA methylation [13]. DNA methylation of leptin and adiponectin promoters in obese children is associated with BMI, dyslipidemia, and insulin resistance [14]. These observations support the hypothesis that epigenetic modifications might underpin the development of obesity and related metabolic disorders. Hypermethylation of the pro-opiomelanocortin and serotonin transporter genes has been positively associated with childhood or adult obesity [15]. HFD changes the methylation status of *Casp1* and *Ndufb9* genes in obese mice, which are related to liver lipid metabolism and liver steatosis [16]. In addition, the leptin promoter was hypermethylated and *Ppar-α* promoter was hypomethylated in oocytes of mice fed with HFD, and the same changes were also observed in the liver of female offspring [17]. However, few studies have reported the changes in perirenal adipose tissue methylation profile in HFD-induced obese rabbits.

To further understand the epigenetic mechanisms influencing fat metabolism in obese rabbits, we investigated the role of DNA methylation in perirenal adipose tissue by sequencing and analyzing DNA methylation libraries from rabbits fed a standard normal diet (SND) and a high-fat diet (HFD).

## 2. Materials and Methods

### 2.1. Animals

A total of 24 female Tianfu black rabbits from a strain breed at the Sichuan Agricultural University in China were randomly divided into two groups and fed either a standard normal diet (SND) or a high-fat diet (HFD; 10% lard was added to the standard normal diet) for four weeks. The composition and nutrient content of the standard normal diet (SND) and the high-fat diet (HFD) were described in our previous report [18]. At the beginning of the trial, all rabbits were 35 days of age and housed individually in a clean iron cage (600 × 600 × 500 mm) and kept in an environmentally controlled room. Rabbits were free to access water and fed twice a day. At the end of the trial, rabbits were screened for obesity using the body mass index (BMI; BMI = bodyweight (kg)/height^2^ (m)), and three rabbits from each group meeting the experimental requirements were selected for sampling. All experimental protocols were performed under the direction of the Institutional Animal Care and Use Committee from the College of Animal Science and Technology, Sichuan Agricultural University, China (DKY-B2019202015, 5 December 2019).

### 2.2. DNA Extraction

Perirenal adipose tissue samples were collected immediately after rabbits were euthanized (shock and bleed treatment). Tissue blocks were placed in 4 mL EP tubes and stored in a −80 °C freezer. Total DNA from perirenal adipose tissue was extracted using a commercial TIANamp Genomic DNA extraction kit (Tiangen, Beijing, China), following the manufacturer’s instructions. Subsequently, the purity and concentration of DNA were assessed by Agilent 2100 Bioanalyzer (Agilent Technologies, Carlsbad, CA, USA), and only DNA meeting quality criteria (thresholds: A_260_/A_280_ ≈ 1.8; concentration ≥ 200 ng/μL) was used for the trial.

### 2.3. DNA Methylation Library Construction and Sequencing

To identify genome-wide DNA methylation changes in perirenal adipose tissue induced by HFD, two libraries were constructed by pooling the DNA samples from three SND rabbits and three HFD rabbits. Briefly, DNA was fragmented by sonication to 100 to 500 bp fragments. The fragments were end-repaired using T4 DNA polymerase and Klenow enzyme and adaptors were ligated after generating 3’dA overhangs. Bisulfite treatment was conducted using the ZYMO EZ DNA Methylation-Gold kit (Zymo Research, Orange, CA, USA), following the manufacturer’s protocol. After desalting, fragments of sizes ranging from 220 to 320 bp were isolated using a 15% PAGE gel and amplified by adaptor-mediated PCR. Lastly, the libraries were sequenced using the Illumina HiSeq 4000 platform (Illumina, San Diego, CA, USA) by Chengdu Life Baseline Technology Corporation, China.

### 2.4. Processing and Comparison of Sequencing Data

By removing adapter sequences and low-quality reads containing more than 50% low-quality bases (quality score < 5), clean reads were retained. Clean reads were aligned to the rabbit reference genome (GCF_000003625.3) with software BSMAP 2.90 (http://code.google.com/p/bsmap). Two forward strands, i.e., BSW (++) and BSC (−+) were used as references. The accuracy of DNA methylation detection depends on the conversion efficiency of cytosine, and the incomplete transformation of cytosine in sequences may lead to false-positive results. Here, lambda phage DNA was used as a control group to calculate the bisulfite conversion rate.

### 2.5. Methylation Site Detection

The methylation C sites were determined using the method described in a previous study [19]. Briefly, a binomial distribution test was performed for methylated reads number and non-methylated reads number at C sites. C sites were identified as the methylation C sites when the number of reads was greater than or equal to the binomial distribution expected value and the total effective coverage was greater than or equal to four.

### 2.6. Methylation Level Analysis

The average genome-wide methylation level reflects the overall characteristics of the methylation pattern of the genome. DNA methylation occurs in three sequence contexts: CG, CHG, and CHH (H = A, C, or T). The average methylation levels of CG, CHG, and CHH were calculated based on the percentage of methylated cytosine in the entire genome, chromosome, and genomic functional elements. For each type of sequence (CG, CHG, and CHH), the average methylation level was calculated according to the following formula: the average methylation level = methylated reads / (methylated reads + non-methylated reads) × 100%. To assess the association between sequence characteristics and methylation bias, we calculated the methylation percentage of nine bases upstream and downstream of the methylation site.

### 2.7. Searching for Differentially Methylated Regions (DMRs)

DMRs were identified using the option of a sliding window method. Briefly, the sliding windows, which were used for further analysis, had to meet the following criteria: (a) the depth in each cytosine should be more than four in each sample, and each C site should cover at least four methylation reads; (b) the number of selected cytosine should be larger than five; (c) after calculating mean methylation level of each sample, the fold change of mean methylation level between the two samples should be larger than two. After repeating extension steps, the merged regions with *p* < 0.05 were defined as DMRs.

### 2.8. Functional Enrichment Analysis of Differentially Methylated Genes

To explore the role of epigenetic variation in biological processes and pathways, online software DAVID Bioinformatics Resources 6.7 (http://david.abcc.ncifcrf.gov/home.jsp) was used to perform Gene Ontology (GO) terms and KEGG (Kyoto Encyclopedia of Genes and Genomes) pathway enrichment analysis of DMRs-associated genes. GO analysis can be used to identify the performance of the gene product and contains three types of information: cellular component, molecular function, and biological processes. KEGG is the main public database that integrates the genome, chemistry, and system function information, particularly the set of genes associated with the systemic functions of cells, organisms, and ecosystems. Differences were considered to be statistically significant at *p* < 0.05.

## 3. Results

### 3.1. Quality Assessment of Sequencing Data

After raw reads were processed, a total of 1,221,455,488 clean reads were obtained from methylation sequencing libraries (Table S1). The clean reads were mapped to the rabbit reference genome, and the mapping rate was 84.910% in the SND group and 84.730% in the HFD group, respectively. The bisulfite conversion rate was 99.550% for SND, and 99.520% for HFD. In addition, the effective coverage rate of C base in each chromosome ranged from 89.892% to 97.577% and ranged from 91.822% to 97.804% in different functional genomic elements (Tables S2 and S3).

### 3.2. Methylation Level Analysis

Genome-wide methylation level analysis showed that the methylation level of C, CHG, and CHH in the HFD group was higher than in the SND group but the CG methylation level in the HFD group was lower than in the SND group (Table S4). Results of the methylation level C, CG, CHG, and CHH on different chromosomes are shown in Table S5. The greatest differences in C, CG, CHG, and CHH between the two groups were found on chromosome 20, chromosome X, chromosome 1, and chromosome 11, reaching 0.569%, 2.736%, 0.056%, and 0.047%, respectively. In addition, we classified the various functional genomic elements into promoter, CDS, intron, mRNA, downstream, CpGIsland, ncRNA, and transposons. Compared with the SND group, the methylation level of C, CHG, and CHH in each functional genomic element was increased in the HFD group (Table S6). However, based on comparison with the SND group, promoter, intron, mRNA, downstream, and ncRNA methylation levels were decreased in CG, and only CDS, CpGIsland, and transposons methylation levels were increased in CG.

### 3.3. Genome-Wide Characteristics of Methylated C Bases

**Figure 1:**
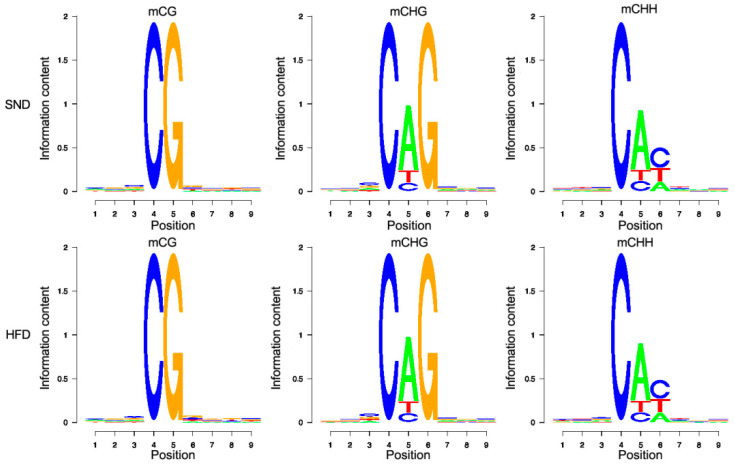
Genome-wide characteristics of methylated C bases. Sequence characteristics of bases near mCG, mCHG, and mCHH in the SND and HFD group.

The percentage of methylated C bases in CG were highest, reaching 94.795% (SND) and 94.843% (HFD) but rarely cytosine methylation was found in CHH and CHG. In addition, we calculated the methylation percentage of nine bases (methylated C at the fourth base) upstream and downstream of the methylated site. As shown in Figure 1, CG, CAG, and CAC were the most likely sites to be methylated in both SND and HFD groups.

### 3.4. Analysis of Differentially Methylated Regions (DMRs)

**Figure 2:**
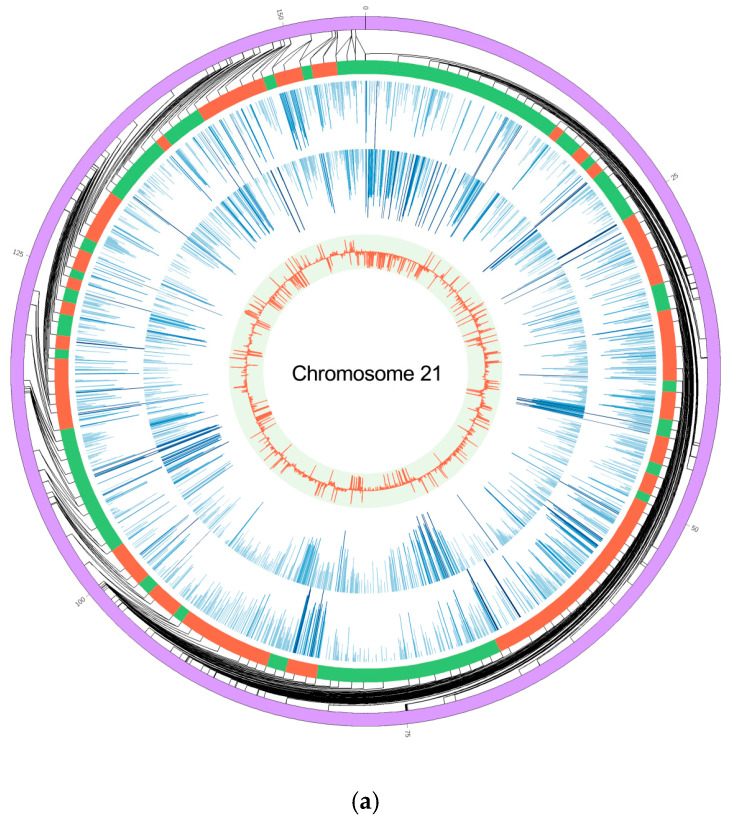

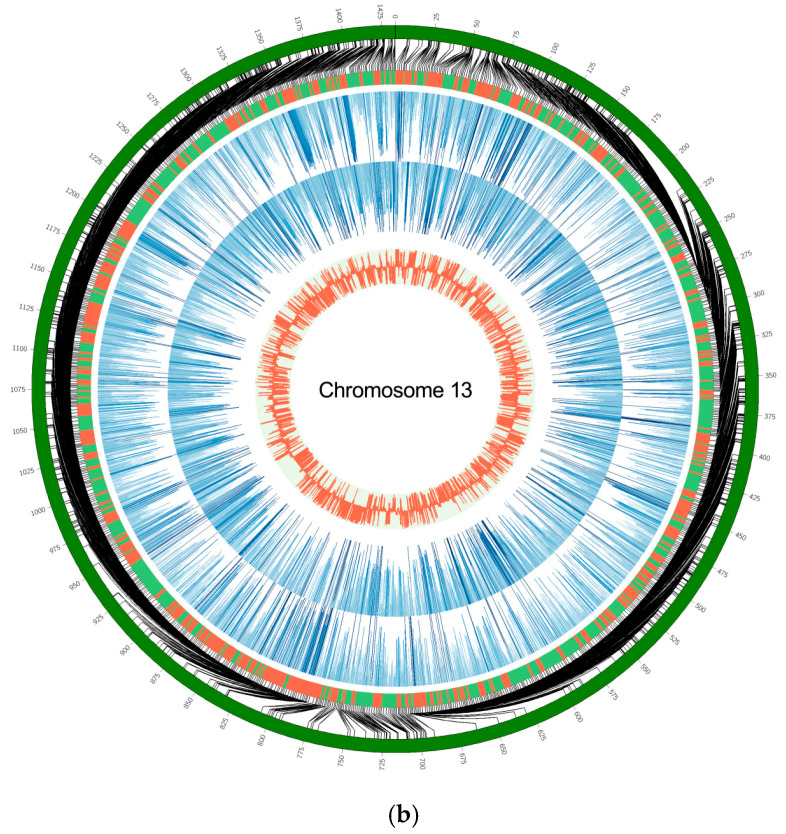
Analysis of differentially methylated regions (DMRs). Two chromosomes with the least (**a**) and the most (**b**) amount of DMRs. The outer ring represents the position of the genomic chromosome; the second circle is the DMRs region: the red area represents the higher methylation level of HFD compared to the SND group and the green area represents the lower methylation level of HFD compared to the SND group; the third circle represents the methylation rate of each site of sample HFD; the fourth circle represents the methylation rate of each site of sample SND; the fifth circle represents the difference of methylation rate.

A total of 12,230 DMRs were identified in the genome of the HFD group compared to the SND group. Chromosome 21 was the chromosome with the least amount of DMRs and chromosome 13 was the chromosome with the most amount of DMRs (Figure 2a,b). The total length of DMRs in each chromosome is shown in Table S7. In addition, the DMRs were mapped to the gene body and promoter regions, and 2305 (1207 up-regulated, 1098 down-regulated) and 601 (368 up-regulated, 233 down-regulated) methylated genes were obtained, respectively. Some genes involved in adipocyte growth and development have also been identified, including *ACE2*, *AGTR1*, *IGF1R*, and *ACSL4*.

### 3.5. GO and KEGG Enrichment Analysis

**Figure 3:**
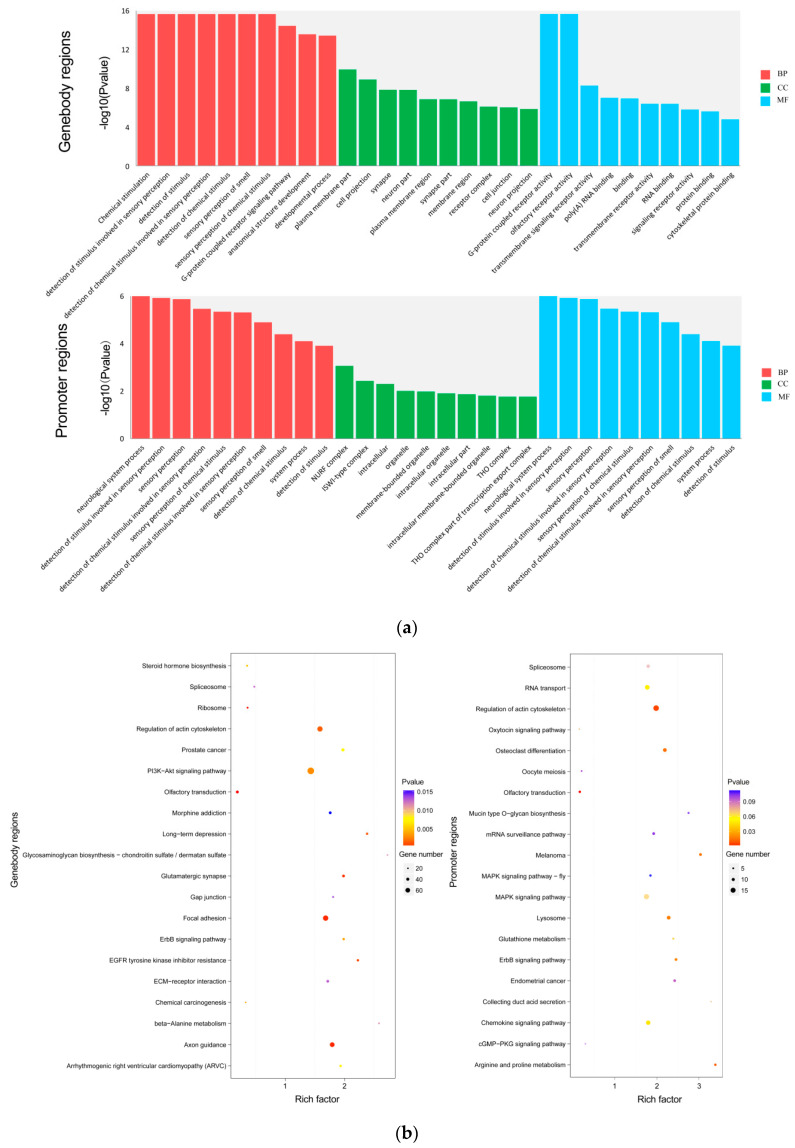
GO and KEGG enrichment analysis. (**a**) GO analysis of the overlapping DMRs-associated genes in the gene body regions and promoter regions. (**b**) KEGG pathway analysis of the overlapping DMRs-associated genes in the gene body regions and promoter regions. Rich factor = (DMRs-associated genes annotation in term/genes annotation in term)/(DMRs-associated genes with KEGG annotation / all genes with KEGG annotation).

To better study the biological functions of the DMRs-associated genes, we used online software DAVID Bioinformatics Resources 6.7 (http://david.abcc.ncifcrf.gov/home.jsp) to carry out gene ontology (GO) terms and KEGG (Kyoto Encyclopedia of Genes and Genomes) pathway enrichment analysis. GO analysis of the overlapping DMRs-associated genes in the gene body regions found a total of 6310 enriched GO terms (4796 biological processes (BP), 579 cellular components (CC), and 935 molecular functions (MF)), of which 12.570% were significantly enriched (*p* < 0.05) (Table S8). The main GO terms involved in overlapping DMRs-associated genes in the gene body regions included the developmental process (GO:0032502), cell differentiation (GO:0030154), and lipid binding (GO:0008289). The top 10 significantly enriched terms in the BP, CC, and MF categories are shown in Figure 3a. The KEGG pathway analysis showed that overlapping DMRs-associated genes in the gene body regions were enriched in 314 pathways including the PI3K-AKT signaling pathway (KO04151), linoleic acid metabolism (KO00591), and pathways for DNA replication (KO03030). Thirty-nine of these pathways (12.420%) were significantly enriched (*p* < 0.05, Table S9). In addition, a scatter analysis was carried out for the 20 most significant pathways to intuitively show the significance of these pathways (Figure 3b).

GO analysis of the overlapping DMRs-associated genes in the promoter regions showed enrichment of 2223 biological processes (BP), 311 cellular components (CC), and 405 molecular functions (MF), of which 173 BP (7.780%), 20 CC (6.430%), and 37 MF (9.140%) were significantly enriched (Table S10). The significantly enriched GO terms mainly include positive regulation of lipid biosynthetic process (GO:0046889), regulation of cholesterol metabolic process (GO:0090181), and regulation of lipid biosynthetic process (GO:0046890). The top 10 significantly enriched GO terms in the BP, CC, and MF categories of GO analysis are shown in Figure 3a. KEGG pathway analysis found 266 enriched pathways including the MAPK signaling pathway (KO04010) (Table S11). The top 20 significantly enriched pathways are presented in Figure 3b.

## 4. Discussion

DNA methylation represents an important epigenetic marker because it is associated with chromosomal structural changes, embryonic development, expression of imprinted genes, and causing corresponding diseases, including X chromosome inactivation and DNA unwinding [20,21,22]. Nowadays, obesity prevention and treatment strategies have been unsuccessful, and DNA methylation is one of the epigenetic modifications associated with obesity [23]. The rabbit is an ideal material to study obesity due to its lipid metabolism and obesity-related clinical manifestations similar to those of humans [24,25]. Thus far, some DNA methylation related studies have been investigated in rabbit models but studies of the changes in perirenal adipose tissue methylation profile in HFD-induced obese rabbits have not been carried out. In this study, DNA methylation patterns were investigated in rabbit models to understand obesity-related DNA methylation changes.

Here, the mapping rates were 84.910% and 84.730% in the SND group and HDF group, respectively. The bisulfite conversion rates were 99.550% (SND) and 99.520% (HFD) in the two groups, which was consistent with previous research, indicating that the libraries were high quality and reliable [26,27]. Methylation is a dynamic process in cells, which can be regulated by methylation and demethylation. The average methylation level of the whole genome reflects the overall characteristics of the genome methylation profile. The results of the genome-wide methylation level analysis in this study were similar to those in mice [28]. The methylation level of CG was higher than the methylation level in C, CHG, and CHH. However, this result is different from that of plant Arabidopsis Thaliana. The plant genome has extensive methylation at the CHG site [29]. CG methylation was maintained by Dnmt1. CHH methylation and some CHG methylation is usually maintained by the activity of the conserved Dnmt3. The high level of CHG methylation seen in Arabidopsis thaliana is maintained by plant-specific methyltransferase [30]. In addition, research on chickens suggests that promoter DNA methylation generally affects chromatin structure and is a signal to inhibit gene transcription, and promoter regions are lowly methylated [31]. Our study also found that promoter regions showed a lower methylation level than other regions. However, a study in mice fed with HFD showed that promoter regions are hypermethylated [32]. Therefore, we hypothesized that differences in methylation level may be species-specific.

The results of genome-wide characteristics of methylated C bases showed that the proportion of mCG was the highest, while the cytosine methylation was low in CHH and CHG. Some studies have shown that no enzyme can maintain mCHG during DNA replication in animals, so the sites of CHG type in animal cells generally show a very low level of methylation. CHH can only rely on the methylation mechanism, so CHH methylation is easily lost in the process of DNA replication and is generally in the state of hypomethylation [33]. The results of this study showed that the characteristics of methylation in the rabbit genome were similar to those in other animals.

DMRs refer to the regions of DNA molecules with different methylation status in two samples. The identification of DMRs is the first step towards the study of DMRs-associated genes [34]. In our study, a total of 2906 DMRs were identified, and 2305 (1207 up-regulated, 1098 down-regulated) and 601 (368 up-regulated, 233 down-regulated) methylated genes were associated with differentially methylated regions. Many genes are related to adipocyte growth and development. For example, as the members of the renin-angiotensin system (RAS), *ACE2* and *AGTR1* were reported to participate in the development and progression of obesity [35,36]. *PPARγ* and *aP2* are important transcription factors in the development and function of the adipose tissue and marker of lipogenesis [37]. Previous studies showed that inhibition of *IGF1R* decreased the expression of PPARγ, thereby inhibiting lipogenesis [38]. Moreover, *ACSL4* plays a role in the regulation of lipid metabolism. *ACSL4* was expressed throughout the entire differentiation process in pig preadipocytes and showed a similar expression trend with lipogenesis-associated genes *PPARγ* and *aP2* [39].

Gene ontology (GO) analysis is a reliable bioinformatics tool for understanding the characteristics of genes and gene products. The significantly enriched terms in the BP, CC, and MF categories indicated the possible roles of the DMRs-associated genes in regulating obesity. The significantly enriched GO terms showed correlation with adipocyte lipid metabolism and metabolisms, such as lipid binding (GO:0008289), positive regulation of lipid biosynthetic process (GO:0046889), regulation of cholesterol metabolic process (GO:0090181), developmental process (GO:0032502), and cell differentiation (GO:0030154). Some terms were related to adipocyte development, including cytoskeletal protein binding (GO:0008092), tubulin binding (GO:0015631), calcium ion binding (GO:0005509). Cytoskeletal remodeling and cell–cell interaction are a necessary step in the transformation of preadipocytes into mature adipocytes, and adipocyte development is dependent on α-tubulin [40,41]. Calcium is a complex mediator in adipogenesis because it regulates numerous cellular processes [42]. Furthermore, other GO items related to hormones and enzymes were also significantly enriched, such as regulation of glucocorticoid secretion (GO:2000849), N-acetyltransferase activity (GO:0008080), and phosphoric diester hydrolase activity (GO:0008081). Increasing evidence suggests that excess glucocorticoids leads to increased fat mass and obesity through the accumulation of adipocytes [43]. Acetyltransferase is a regulator of adipogenesis and lipid metabolism, and its regulatory mechanism is mainly transcription and post-translation modifications [44]. Phosphoric diester hydrolase is a regulator of systemic glucose and insulin homeostasis [45]. Interference of phosphoric diester hydrolase expression in 3T3-L1 adipocytes caused a dramatic decrease in adipocyte differentiation key gene (*PPARγ*, *aP2*) and lipid accumulation [46].

Adipogenesis is a complex process involving an elaborate network of transcription factors and signaling pathways. Results of KEGG analysis showed that DMRs-associated genes were mainly involved in the PI3K-AKT signaling pathway (ko04151), linoleic acid metabolism (KO00591), DNA replication (KO03030), and MAPK signaling pathway (KO04010). The PI3K-AKT signaling pathway is a key regulator in cell proliferation, differentiation, and apoptosis [47]. Activation of the PI3K-AKT signaling pathway promotes the expression of marker genes involved in adipogenesis and glucose uptake [48]. In our study, 70 DMRs-associated genes were enriched in the PI3K-AKT signaling pathway, thereby revealing that these DMRs-associated genes may be essential for adipogenesis. Linoleic acid metabolism (KO00591) is also associated with adipogenesis. Linoleic acid can be converted to the metabolically active arachidonic acid, which has roles in inducing inflammation and adipogenesis. Excessive intake of linoleic acid results in increasing magnitudes of adiposity, inflammatory cytokines, and insulin resistance [49]. In addition, it is becoming clear that DNA replication (KO03030) and the MAPK signaling pathway (KO04010) play an important role in adipocyte growth and development [50,51]. Thus, the results of our study indicate that these DMRs-associated genes might be an important regulator in adipogenesis. However, due to the limitation of experimental conditions, such as pooled samples, only one library per group, sequencing methods, etc., functional verification of these DMRs-associated genes will be important to consider in the future.

## 5. Conclusions

In conclusion, our study indicates that a high-fat diet may affect genes associated with adipogenesis by altering DNA methylation patterns. We identified 2906 methylated genes, of which, *ACE2*, *AGTR1*, *IGF1R*, and *ACSL4* may have a key role in adipogenesis. These genes may be involved in the regulation of adipogenesis through the PI3K-AKT signaling pathway (KO04151), linoleic acid metabolism (KO00591), DNA replication (KO03030), and MAPK signaling pathway (KO04010).

## Supplementary Materials

The following are available online at https://www.mdpi.com/2076-2615/10/12/2213/s1, Table S1: Data output and comparative statistics, Table S2: The effective coverage rate of C base in each chromosome, Table S3: The effective coverage rate of C base in different functional genomic elements, Table S4: Results of genome-wide methylation level analysis, Table S5: Results of the methylation level C, CG, CHG, and CHH on different chromosomes, Table S6: Methylation level in various functional genomic elements between standard normal diet (SND) and high-fat diet (HFD) group, Table S7: DMRs number and length statistics of standard normal diet (SND) and high-fat diet (HFD) group, Table S8: GO analysis of the overlapping DMRs-associated genes in the gene body regions, Table S9: KEGG pathway analysis of the overlapping DMRs-associated genes in the gene body regions, Table S10: GO analysis of the overlapping DMRs-associated genes in the promoter regions, Table S11: KEGG pathway analysis of the overlapping DMRs-associated genes in the promoter regions.
